# Factors Associated with Depression, Anxiety, and Stress in Men and Women: Findings from a Population-Based Study in Iran

**DOI:** 10.34172/aim.2022.85

**Published:** 2022-08-01

**Authors:** Fahimeh Mehrabi, Parisa Amiri, Parisa Naseri, Fereidoun Azizi

**Affiliations:** ^1^Research Center for Social Determinants of Health, Research Institute for Endocrine Sciences, Shahid Beheshti University of Medical Sciences, Tehran, Iran; ^2^Endocrine Research Center, Research Institute for Endocrine Sciences, Shahid Beheshti University of Medical Sciences, Tehran, Iran

**Keywords:** Anxiety, Behavioral characteristics, Depression, Socio-demographic characteristics, Stress

## Abstract

**Background:**

This study aimed to investigate the relationship between personal and clinical characteristics of adults in the Tehran Lipid and Glucose Study (TLGS) with depression, anxiety, and stress.

**Methods:**

Data of 2272 adults participating in the 6^th^ phase of TLGS were used for univariate analysis to investigate the association between socio-demographic, behavioral, and clinical characteristics and participants’ emotional states. Thereupon, the predictors with a *P* value<0.20, at least for one of depression, anxiety, and stress in the primary analysis, were included in the model for multivariate modeling.

**Results:**

The mean age of participants was 47.23±14.87. The mean scores of depression, anxiety, and stress were higher in women (*P* value:<0.001,<0.001, and 0.004) than in men. Higher age was associated with lower anxiety (β=-0.04, *P*=0.004) and stress (β=-0.13, *P*<0.001) in men, but only lower stress in women (β=-0.07, *P*=0.001). Highly educated participants experienced lower depression (β=-2.26, *P*=0.01, β=-2.26, *P*=0.003). Although married men robustly experienced lower depression than others (β=-1.69, *P*=0.009), a less powerful relationship existed between being married and depression in women (β=-1.37, *P*=0.05). All cigarette smokers experienced higher depression, anxiety, and stress, but only female hookah smokers had higher anxiety and stress. Physical activity and chronic disorders had no relationship with emotional states. Obesity was associated with stress in women (β=1.95, *P*=0.001).

**Conclusion:**

This study provides an update on factors associated with mental health outcomes in a large general population. Examining the factors associated with depression, anxiety, and stress through a sex-sensitive lens could help clinicians diagnose and plan the best preventive and therapeutic approach.

## Introduction

 Depression, anxiety, and stress have been the most common mental health conditions over the past decades. Alongside the rising global rates of these psychological outcomes,^1^ their burden and prevalence have also increased in the Middle East region.^2,3^ Likewise, the high prevalence of depression (44%), anxiety (42%), and stress (40%) symptoms in Iran intensify concerns about their far-reaching consequences on health, productivity, and quality of life that could lead to suicide at worst.^4-8^

 The tangible effects of depression, anxiety, and stress on human life in the modern era are undeniable. Accordingly, many studies have been conducted worldwide for more than a century to understand these common mental health outcomes. Although there are much controversial data about their different dimensions, the gender difference in depression and anxiety with higher prevalence in women is one of the most robust psychological phenomena that is universally accepted.^9,10^ Following this fact, many questions were raised about sex differences that led to recognizing the distinct expressions of depression and different coping mechanisms in men and women.^11^ Also, increased stress vulnerability in women and different anxiety treatment responses in both sexes have been noticed.^12^ Still, the role of potential factors associated with depression, anxiety, and stress in each sex remained unclear.

 In general, some socio-demographic, behavioral, and clinical factors, including age,^9,13^ marital status,^14^ educational level,^15^ health-related behaviors,^16^ obesity,^17,18^ and chronic disorders^19^ are among suggested determinants of common mental health conditions in previous studies.^20-22^ However, there are a few issues to consider about these factors: Firstly, sex differences in socio-demographic and behavioral characteristics have not been studied as much as the biological characteristics affecting men’s and women’s mental health.^23,24^ Secondly, despite the importance of cultural issues and the need for up-to-date reports on common psychological conditions from the transitional communities in the Middle East region, studies in this field have been conducted mostly in developed countries. Thirdly, although there is relative agreement on the effect of the above-mentioned determinants, many contradictory results have been reported from different parts of the world. In the latter case, few studies have examined the association of several potential determinants with the three common psychological states of depression, anxiety, and stress in the general population comprehensively.

 Regarding the multifactorial nature of common mental health outcomes (depression, anxiety, and stress), the impact of sex and cultural context, and their high prevalence, evaluating the potential determinants and comparing them in men and women seems necessary to yield a comprehensive picture. The current study aimed to address the socio-demographic, behavioral, and clinical determinants of depression, anxiety, and stress and compare them among men and women residents of Tehran to provide a multi-dimensional view and help clinicians in their therapeutic approaches.

## Materials and Methods

###  Study Population

 The participants of the current study were recruited from the Tehran Lipid and Glucose Study (TLGS), an ongoing community-based study aimed at determining cardiovascular diseases and related risk factors as well as preventing non-communicable diseases among the residents of district 13 of Tehran. The first phase of the TLGS was conducted during 1999–2002 and then followed triennially. More study details have been published previously.^25^ In the current study, from 9,708 individuals (aged ≥ 20 years) who participated in the 6^th^ phase of the TLGS (2014–2016), 2,583 individuals randomly completed the DASS-21 (Depression, Anxiety, and Stress Scale) questionnaire, and 311 participants with missing data on socio-demographic*, behavioral, and clinical information were excluded. The final sample, including* 2272 adults with complete data on depression, anxiety, and stress, was considered for analysis.

###  Socio-demographic, Behavioral, and Clinical Information 

 Socio-demographic (age, education level, employment, and marital status), behavioral (smoking status and physical activity level), and clinical information (chronic disease and obesity) of the participants was obtained from the TLGS data bank. Education level was divided into three categories: 1) primary: illiterate or less than high school; 2) secondary: high school; 3) higher: higher than high school degree. In terms of smoking status, individuals were classified as smokers and non-smokers in cigarette and hookah subcategories. Physical activity levels were assessed using the Iranian version of the Modifiable Activity Questionnaire (MAQ). The frequency and duration of each leisure time and work physical activity (standing, housework, and work activities more intense than standing) were calculated. Then, they were multiplied by the weight and the metabolic equivalent task (MET) of the particular act to determine the energy expenditure. Total physical activity was calculated and classified into the following categories: 1) low ( < 600), 2) moderate (600–3000), and 3) high ( ≥ 3000) physical activity by adding the energy expenditure of each domain.^26^ Chronic disease was defined as having diabetes mellitus, hypertension, cardiovascular disease, and cancer. Obesity was defined as body mass index (BMI) ≥ 30.0 kg/m^2^.

###  Depression, Anxiety, and Stress Symptoms

 The participants’ mental health was assessed by the Persian version of DASS-21,^27^ which is a short form of the 42-item scale. This self-report questionnaire (DASS-21) has three 7-item subscales with similar content. Scores for each subscale are calculated as the sum of responses to the relevant items, and a higher score in each subscale indicates a more severe condition.^28^ The validity and reliability of this questionnaire have been published previously. For the total score of DASS-21, the Cronbach alpha was 0.94, and for each of the depression, anxiety, and stress scales, the values were 0.85, 0.85, and 0.87, respectively.^27^

###  Statistical Analysis

 Continuous and categorical variables were reported as mean ± SD and frequency (percent), respectively. First, a univariate analysis was conducted to examine the association between each predictor and the participants’ emotional states. Thereupon, the predictors with a *P* value < 0.20, at least for one of the emotional state dimensions in the univariate analysis, were included in the model for multivariate analysis. Two-sided *P* values less than 0.05 were considered statistically significant. Data analyses were conducted using IBM SPSS Statistics 23.

## Results

 The participants were 2272 men and women (55.72% women) with a mean age of 48.99 ± 17.38 and 49.11 ± 16.06, respectively, with a range of 20 to 87 years. Socio-demographic, behavioral, and clinical characteristics are represented in Table 1. The majority of participants had a high school degree (51% of men and 49.5 % of women) and were married (81.6% of men and 76.1% of women). The employment rate varied greatly between men and women (74.3% vs. 17.9%). Most of the participants were non-smokers and non-obese with high physical activity and no chronic disease.

**Table 1:** Distribution of Socio-demographic, Behavioral, and Clinical Characteristics of Participants (n= 2272)

	**Men** **(n=1006)**	**Women** **(n=1266)**	***P *** **Value**
**Socio-demographic Characteristics**			
Age (y)	48.99 ± 17.38	49.11 ± 16.06	< 0.001
Age (categories)			< 0.001
20–35	271 (26.9)	325 (25.7)	
36–50	306 (30.4)	416 (32.9)	
> 50	429 (42.6)	525 (41.5)	
Education level			< 0.001
Primary	93 (9.2)	239 (18.9)	
Secondary	513 (51)	627 (49.5)	
Higher	400 (39.8)	400 (31.6)	
Employment status			< 0.001
Employed	747 (74.3)	226 (17.9)	
Unemployed	259 (25.7)	1040 (82.1)	
Marital status			< 0.001
Single	171 (17)	158 (12.5)	
Married	821 (81.6)	964 (76.1)	
Divorced/Widowed	14 (1.4)	144 (11.4)	
**Behavioral Characteristics**			
Cigarette smoking			< 0.001
Non-smoker	755 (75)	1217 (96.1)	
Smoker	251 (25)	49 (3.9)	
Hookah smoking			< 0.001
Non-smoker	787 (78.2)	1173 (92.7)	
Yes	219 (21.8)	93 (7.3)	
Physical activity			< 0.001
Low	382 (38)	365 (28.8)	
Moderate	212 (21.1)	415 (32.8)	
High	412 (41)	486 (38.4)	
**Clinical Characteristics**			
Chronic diseases			< 0.001
No	559 (55.6)	678 (53.6)	
Yes	447 (44.4)	588(46.4)	
Weight status			< 0.001
Non-obese	748 (74.4)	814 (64.3)	
Obese	258 (25.6)	452 (35.7)	

Continuous and categorical data are presented as mean ± SD and frequency (%), respectively.

**Figure 1:**
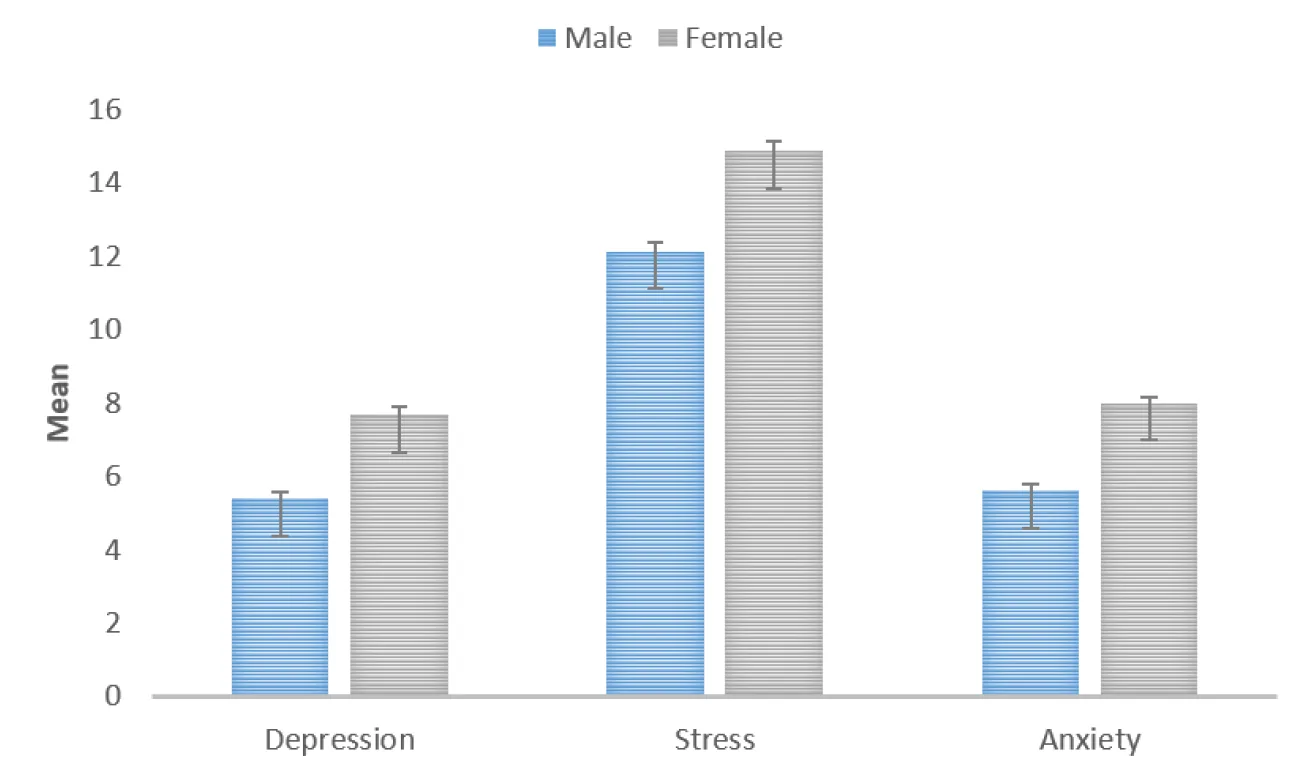
The depression, stress, and anxiety means among men and women

 As Figure 1 shows, the mean scores of depression, anxiety, and stress were significantly higher in women (*P* < 0.001, < 0.001, and 0.004) than men. The sex-specific results of the univariate analysis conducted to examine the association between each predictor and participants’ emotional state are shown in Table 2. The multivariate analysis results, which include the adjusted effects of selected variables on men and women’s depression, anxiety, and stress, are presented in Tables 3 and 4, respectively. In men and women, higher age was significantly associated with lower depression (β: -0.07, *P* < 0.001; β: -0.06, *P* = 0.01, respectively), anxiety (β: -0.08, *P* < 0.001; β: -0.07, *P* = 0.001, respectively), and stress (β: -0.14, *P* < 0.001; β: -0.12, *P* < 0.001, respectively). Men and women who had high school degrees experienced lower depression (β: -1.55, *P* = 0.003; β: -1.55, *P* = 0.02, respectively) and anxiety (β: -2.79, *P* = 0.03; β: -2.29, *P* = 0.004, respectively) in comparison to their counterparts with primary education level. Likewise, highly educated men had significantly lower depression, anxiety, and stress; and highly educated women reported lower depression and stress. Unemployed men had higher depression (β: 1.67, *P* < 0.001), anxiety (β: 1.53, *P* < 0.001), and stress (β: 1.64, *P* < 0.001) compared to employed men. Married men experienced lower depression (β: -0.99, *P* = 0.05), and the divorced/widowed reported higher anxiety (β: 2.16, *P* value: 0.003) than the singles. Regardless of sex, all cigarette smokers experienced higher depression, anxiety, and stress, but only female hookah smokers had higher anxiety (β: 1.89, *P* = 0.02) and stress (β: 2.47, *P* = 0.02). Physical activity level had no relationship with emotional states in either gender. Both sexes with chronic disease reported higher anxiety. Obesity had a significant association with depression (β: 0.72, *P* = 0.03), anxiety (β: 0.81, *P* = 0.009), and stress (β: 1.30, *P* = 0.003) in men but only with stress (β = 1.95, *P* = 0.001) in women.

**Table 2:** Unadjusted Associations between Participants’ Characteristics and their Emotional State

**Variables**	**Men (n=1006)**						**Women (n=1266)**					
	**Depression**		**Anxiety**		**Stress**		**Depression**		**Anxiety**		**Stress**	
	**β (SE)**	***P***	**β (SE)**	***P***	**β (SE)**	***P***	**β (SE)**	***P***	**β (SE)**	***P***	**β (SE)**	***P***
**Socio-demographic Characteristics**												
Age	-0.42 (0.01)	< 0.001	-0.03 (0.01)	0.004	-0.10 (0.02)	< 0.001	0.02 (0.02)	0.34	0.006 (0.01)	0.67	-0.06 (0.02)	0.001
Education level*												
Primary	(Ref.)		(Ref.)		(Ref.)	(Ref.)	(Ref.)	(Ref.)	(Ref.)	(Ref.)	(Ref.)	(Ref.)
Secondary	-0.005 (0.68)	0.99	0.40 (0.63)	0.52	1.94 (0.94)	0.04	-1.23 (0.60)	0.04	-0.63 (0.54)	0.24	0.48 (0.71)	0.50
Higher	-0.53 (0.70)	0.44	-0.16 (0.64)	0.80	1.42 (0.96)	0.14	-2.62 (0.64)	< 0.001	-1.47 (0.58)	0.01	-0.20 (0.76)	0.79
Employment												
No	(Ref.)		(Ref.)		(Ref.)	(Ref.)	(Ref.)	(Ref.)	(Ref.)	(Ref.)	(Ref.)	(Ref.)
Yes	0.54 (0.43)	0.21	-0.18 (0.40)	0.65	-0.91 (0.59)	0.13	1.19 (0.57)	0.04	0.88 (0.51)	0.08	-0.14 (0.67)	0.84
Marital status												
Single	(Ref.)		(Ref.)		(Ref.)	(Ref.)	(Ref.)	(Ref.)	(Ref.)	(Ref.)	(Ref.)	(Ref.)
Married	-2.42 (0.48)	< 0.001	-0.79 (0.44)	0.07	-2.55 (0.66)	< 0.001	-0.83 (0.65)	0.21	0.55 (0.59)	0.35	-0.69 (0.78)	0.38
Divorced/Widowed	0.70 (1.68)	0.68	-0.87 (1.56)	0.58	-2.96 (2.32)	0.20	1.25 (0.89)	0.16	1.81 (0.80)	0.02	-1.42 (1.05)	0.18
**Behavioral Characteristics**												
Cigarette smoking												
Non-smoker	(Ref.)		(Ref.)		(Ref.)	(Ref.)	(Ref.)	(Ref.)	(Ref.)	(Ref.)	(Ref.)	(Ref.)
Smoker	1.39 (0.43)	0.001	1.67 (0.39)	< 0.001	2.56 (0.58)	< 0.001	2.91 (1.11)	0.009	3.14 (0.99)	0.002	4.66 (1.30)	< 0.001
Hookah smoking												
Non-smoker	(Ref.)		(Ref.)		(Ref.)	(Ref.)	(Ref.)	(Ref.)	(Ref.)	(Ref.)	(Ref.)	(Ref.)
Smoker	0.77 (0.49)	0.109	0.82 (0.44)	0.065	2.10 (0.65)	0.001	1.39 (0.82)	0.09	2.05 (0.73)	0.005	3.70 (0.96)	< 0.001
Physical activity**												
Low	(Ref.)		(Ref.)		(Ref.)	(Ref.)	(Ref.)	(Ref.)	(Ref.)	(Ref.)	(Ref.)	(Ref.)
Moderate	-0.10 (0.51)	0.85	-0.13 (0.47)	0.79	-0.35 (0.71)	0.62	-1.20 (0.57)	0.03	-0.9 (0.51)	0.86	-1.04 (0.67)	0.12
High	-0.65 (0.43)	0.13	-0.42 (0.40)	0.29	-0.55 (0.60)	0.36	-1.0 (0.54)	0.07	0.10 (0.49)	0.83	-0.78 (0.64)	0.23
**Clinical Characteristics**												
Chronic diseases												
No	(Ref.)		(Ref.)		(Ref.)	(Ref.)	(Ref.)	(Ref.)	(Ref.)	(Ref.)	(Ref.)	(Ref.)
Yes	-0.65 (0.39)	0.10	0.13 (0.36)	0.72	-0.84 (0.54)	0.12	0.40 (0.45)	0.38	0.84 (0.40)	0.04	-0.63 (0.54)	0.24
Obesity												
No	(Ref.)		(Ref.)		(Ref.)	(Ref.)	(Ref.)	(Ref.)	(Ref.)	(Ref.)	(Ref.)	(Ref.)
Yes	0.37 (0.44)	0.40	0.70 (0.40)	0.08	0.79 (0.60)	0.19	0.97 (0.47)	0.04	0.85 (0.42)	0.04	1.07 (0.55)	0.05

*Primary: illiterate or less than high school; secondary: high school; 3) higher: higher than high school degree; **low physical activity: 600 MET/Week, Moderate physical activity: > 600 and ≤ 3000 MET/Week, High physical activity: > 3000 MET/Week.

**Table 3:** Adjusted Associations between Men’s Characteristics and their Emotional State

**Variables**	**Depression**		**Anxiety**		**Stress**	
	**β (SE)**	***P *** **Value**	**β (SE)**	***P *** **Value**	**β (SE)**	***P *** **Value**
Age	-0.07 (0.01)	< 0.001	-0.08 (0.01)	< 0.001	-0.14 (0.01)	< 0.001
Education level						
Primary	(Ref.)	(Ref.)	(Ref.)	(Ref.)	(Ref.)	(Ref.)
Secondary	-1.55 (0.51)	0.003	-1.01 (0.47)	0.03	-0.05 (0.64)	0.10
Higher	-2.79 (0.58)	< 0.001	-1.85 (0.53)	0.01	-2.41 (0.73)	0.001
Employment						
Yes	(Ref.)	(Ref.)	(Ref.)	(Ref.)	(Ref.)	(Ref.)
No	1.67 (0.34)	< 0.001	1.53 (0.31)	< 0.001	1.64 (0.43)	< 0.001
Marital status						
Single	(Ref.)	(Ref.)	(Ref.)	(Ref.)	(Ref.)	(Ref.)
Married	-0.99 (0.50)	0.05	0.59 (0.46)	0.20	0.22 (0.64)	0.72
Divorced/Widowed	1.10 (0.81)	0.17	2.16 (0.74)	0.003	0.65 (1.02)	0.52
Cigarette smoking						
Non-smoker	(Ref.)	(Ref.)	(Ref.)	(Ref.)	(Ref.)	(Ref.)
Smoker	0.94 (0.48)	0.05	0.89 (0.44)	0.04	1.40 (0.60)	0.02
Hookah smoking						
Non-smoker	(Ref.)	(Ref.)	(Ref.)	(Ref.)	(Ref.)	(Ref.)
Smoker	-0.01 (0.49)	0.97	0.56 (0.44)	0.21	0.78 (0.61)	0.20
Physical activity						
Low	(Ref.)	(Ref.)	(Ref.)	(Ref.)	(Ref.)	(Ref.)
Moderate	-0.40 (0.40)	0.31	0.21 (0.36)	0.56	-0.34 (0.50)	0.49
High	-0.65 (0.36)	0.07	0.05 (0.33)	0.86	-0.50 (0.46)	0.27
Chronic disease						
No	(Ref.)	(Ref.)	(Ref.)	(Ref.)	(Ref.)	(Ref.)
Yes	0.15 (0.37)	0.67	0.93 (0.34)	0.006	0.78 (0.47)	0.09
Obesity						
No	(Ref.)	(Ref.)	(Ref.)	(Ref.)	(Ref.)	(Ref.)
Yes	0.72 (0.34)	0.03	0.81 (0.31)	0.009	1.30 (0.43)	0.003

The predictors with a *P *value < 0.20, at least for one of depression, anxiety, and stress in the univariate analysis, were included in the multivariate model. *Primary: illiterate or less than high school; secondary: high school; 3) higher: higher than high school degree; **Low physical activity: 600 MET/Week, Moderate physical activity: > 600 and ≤ 3000 MET/Week, High physical activity: > 3000 MET/Week

**Table 4:** Adjusted Associations between Women’s Characteristics and their Emotional State

**Variables**	**Depression**		**Anxiety**		**Stress**	
	**β (SE)**	***P***	**β (SE)**	***P***	**β (SE)**	***P***
Age	-0.06 (0.2)	0.01*	-0.07 (0.02)	0.001	-0.12 (0.02)	< 0.001
Education level						
Primary	(Ref.)	(Ref.)	(Ref.)	(Ref.)	(Ref.)	(Ref.)
Secondary	-1.55 (0.70)	0.02	-2.29 (0.80)	0.004	-1.09 (0.83)	0.19
Higher	-3.58 (0.89)	< 0.001	-1.01 (0.63)	0.11	-3.06 (1.06)	0.004
Employment						
Yes	(Ref.)	(Ref.)	(Ref.)	(Ref.)	(Ref.)	(Ref.)
No	0.17 (0.67)	0.79	0.11 (0.60)	0.84	-0.78 (0.79)	0.32
Marital status						
Single	(Ref.)	(Ref.)	(Ref.)	(Ref.)	(Ref.)	(Ref.)
Married	-0.75 (0.77)	0.33	0.69 (0.69)	0.32	0.53 (0.92)	0.56
Divorced/Widowed	0.69 (1.07)	0.51	1.69 (0.96)	0.08	0.10 (1.27)	0.93
Cigarette smoking						
Non-smoker	(Ref.)	(Ref.)	(Ref.)	(Ref.)	(Ref.)	(Ref.)
Smoker	2.31 (1.22)	0.05	2.37 (1.10)	0.03	3.77 (1.45)	0.01
Hookah smoking						
Non-smoker	(Ref.)	(Ref.)	(Ref.)	(Ref.)	(Ref.)	(Ref.)
Smoker	0.99 (0.92)	0.27	1.89 (0.83)	0.02	2.47 (1.09)	0.02
Physical activity						
Low	(Ref.)	(Ref.)	(Ref.)	(Ref.)	(Ref.)	(Ref.)
Moderate	-0.92 (0.56)	0.10	0.05 (0.52)	0.91	-0.95 (0.68)	0.16
High	-0.92 (0.57)	0.11	0.08 (0.50)	0.87	-1.02 (0.66)	0.12
Chronic disease						
No	(Ref.)	(Ref.)	(Ref.)	(Ref.)	(Ref.)	(Ref.)
Yes	0.08 (0.55)	0.88	0.98 (0.49)	0.04	0.34 (0.65)	0.6
Obesity						
No	(Ref.)	(Ref.)	(Ref.)	(Ref.)	(Ref.)	(Ref.)
Yes	0.75 (0.49)	0.12	0.79 (0.44)	0.07	1.67 (0.59)	0.005

The predictors with a *P* value < 0.20, at least for one of depression, anxiety, and stress in the univariate analysis, were included in the multivariate model. *Primary: illiterate or less than high school; secondary: high school; 3) higher: higher than high school degree; **Low physical activity: 600 MET/Week, Moderate physical activity: > 600 and ≤ 3000 MET/Week, High physical activity: > 3000 MET/Week.

## Discussion

 The current study aimed to address some socio-demographic, behavioral, and clinical determinants of depression, anxiety, and stress in Tehran’s adult residents and compare them between men and women. Our results showed that, in general, women experienced higher levels of depression, anxiety, and stress than men. Regarding the relationship between socio-demographic characteristics and common psychological conditions, aging had significant associations with reduced depression, anxiety, and stress in both sexes. Higher education was associated with reduced depression, anxiety, and stress in men; and lower depression and stress in women. Unemployed men experienced higher depression, anxiety, and stress than the employed. Married men had lower depression, while the divorced/widowed reported higher anxiety levels compared to their single counterparts. In terms of health-related behavioral factors, cigarette smoking was associated with increased depression, anxiety, and stress in both sexes. However, there was a significant relationship between hookah smoking with increased anxiety and stress only in women. Among clinical characteristics, chronic disease had a significant relationship with higher anxiety levels in both sexes. Obesity had a significant positive association with depression, anxiety, and stress in men and only stress in women.

###  Sex Differences in Common Mental Health Outcomes

 Our findings regarding higher means of depression, anxiety, and stress scores in women compared to men are consistent with the literature. In fact, one of the most robust phenomena in psychology is the gender difference in depression and anxiety, to a greater extent in women. Previous studies have considered many possibilities to justify this difference. A recent narrative review has comprehensively presented evidence for biological, affective, and cognitive vulnerabilities in women that result in more depression. Similarly, the undeniable impact of exposure to stress and sociocultural factors has been discussed.^9^ The prevalence of anxiety has also been reported two to three times higher in women than men worldwide. Biological differences, such as brain structure and female hormonal fluctuations during the various phases of the menstrual cycle, play a significant role in the higher prevalence and complexity of these disorders in women.^10^

###  Socio-demographic Characteristics

 Our findings showed that depression, anxiety, and stress decreased with advancing age, just as previous studies showed that aging could be a protective factor against the aforementioned psychological conditions.^9,13^ However, it is essential to note the role of age in gender differences in depression and anxiety, which is the gender-specific change in the prevalence of these common psychological conditions throughout the lifespan. While between puberty and menopause, mood and anxiety disorders are more prevalent in women, this difference disappears after menopause.^9,29^ Yet, the decrease in common mental problems with aging should not mislead clinicians to overlook the mood and anxiety disorders and their specific risk factors (e.g., loneliness) in elderly adults^30^ since the association is statistically significant. However, the clinical effect is not much.

 Based on our results, each higher education level in men was significantly associated with lower depression, anxiety, and stress. In women, higher education was associated with a reduction in depression. However, decreased anxiety was associated only with secondary education level and reduced stress with only higher education level. In line with our results, higher education levels have been considered a protective factor against depression because they positively relate to self-efficacy. Also, people with higher education are more likely to have a more fulfilling career.^15^

 Our findings indicated that unemployed men experienced higher depression, anxiety, and stress than employed ones, while no association was found in women. A large body of evidence has mentioned numerous adverse effects of unemployment, such as poor general health and quality of life, depression, anxiety, and mortality regardless of sex.^31,32^ A recent study among the Swedish population, which detected a significant association between unemployment with depression and anxiety, showed greater negative health effects for men.^33^ In line with our findings, another study conducted in southeastern Iran found no difference between employed and housewife women’s quality of life.^34^ Sex differences in this issue are tied to cultural norms. Despite the weakening of the male breadwinning model in many countries, Mediterranean and East Asia countries have started a fragmented trajectory in this direction.^35^ Nevertheless, given the low rate of women’s labor force participation in Middle Eastern countries, it seems that men are still responsible for providing for household expenses.^36^ Thus, being unemployed is not a negative psychological burden for a woman in traditional societies.

 Our results showed that married men had lower depression, while the divorced/widowed reported higher anxiety compared to their single counterparts. The beneficial effects of marriage on physical and mental health have long been established.^14^ In this regard, cultural factors can play an important role, especially in Eastern cultures, where the family formation is significant. A recent study showed negative stereotypes about celibacy among the Turkish population,^37^ and a survey in Pakistan revealed the relationship between fear of negative evaluation and loneliness in single people.^38^ Despite all these, our results did not confirm any connection between marriage and mental health in women. To understand the meaning of this gender difference in the results, we can refer to a study in five cultures which showed that women in all populations have more perceived problems than men.^39^ Another study found that life satisfaction after divorce is higher for women with poor marriage quality.^40^ Therefore, alongside all the positive features mentioned for marriage, special attention should be paid to women’s conditions and psychological needs since factors such as marital conflicts could affect couples’ mental states.^41,42^

 This inconsistency may have been caused by some factors that we did not measure in the current study, but have affected the associations. For instance, burnout, work engagement, occupational stress, and job satisfaction are some work-related variables that also affect individuals’ mental health.^43^

###  Behavioral Characteristics

 Concerning the association of cigarette smoking with depression, anxiety, and stress, regardless of sex, our findings revealed a positively significant relationship. A meta-analysis, which includes longitudinal studies in different populations, finds the results contradictory. However, over a third of the findings showed that continued smoking increases susceptibility to depression and anxiety.^44^ Another sex difference in the current result was that increased anxiety and stress were related to hookah smoking in women but not in men. In line with our findings for women, a recent meta-analysis revealed a significant association between hookah smoking with reduced mental health and poor quality of life.^45^ However, the studies included in the mentioned meta-analysis were not sex-specific, and different tools were used to assess the participants’ mental status. Two separate investigations also reached different hookah smoking motivations among the two genders.^46,47^ Iranian women are not as free as men in cigarette consumption while they could smoke hookah with much less social stigma; at the same time, hookah smoking is more of an entertaining issue for men.^48,49^

 Surprisingly, there was no relationship between physical activity levels and common mental health outcomes in the current study. However, more longitudinal studies showed that higher physical activity could be a protective factor against depression and anxiety.^16,50^ Further time-varying longitudinal investigations seem essential to clarify this controversy.

###  Clinical Characteristics

 Our findings showed a significant relationship between chronic disease and increased anxiety levels. The results of many studies on chronic illness and mental health have also confirmed a relationship. However, some details, such as the relationship’s direction, the type of chronic disease, and the mental state were not the same in different populations.^19,51^ For instance, unlike a stroke, loss of hearing, vision loss, cardiac disease, and chronic lung disease that increase depression, the effects of some other disorders like osteoarthritis, hypertension, and diabetes on mental states are not clear yet.^19^ However, it is essential to note that although many studies have shown the importance of treating depression in patients with chronic illnesses, based on our findings, the development of anxiety treatments for these patients should be a priority.

 Another clinical characteristic related to mental health outcomes considered in the current study was excess weight. Based on our findings, men with obesity reported significantly higher depression, anxiety, and stress levels, while obesity only had a significant association with greater stress in women. Since the relationship between people’s weight status and mental states is complex and influenced by many factors, there are contradictory results on this subject. Some studies have recognized that obesity increases depression and anxiety,^17,18^ and others have found no association,^52^ and gender differences in this issue have been evident. Still inconsistent with the current findings, most previous studies have emphasized that women are more emotionally affected by obesity than men. Mass media and the thin-ideal body image have been known as the main reasons for these adverse mental effects of obesity on women.^53^ On the other hand, cultures are not the same in shaping people’s behavior, and even a lifestyle that leads to obesity in women is encouraged in the Middle East region.^54^

 The current study has some strengths and limitations. This study attempts to evaluate a wide range of sex-specific possible determinants of depression, anxiety, and stress in a large population of Tehrani residents. However, the cross-sectional design of the study precludes causal inferences. In addition, since the sample was from Tehran’s urban population, generalizing the results to a rural population is not reasonable.

 In conclusion, the mean scores of depression, anxiety, and stress were higher in women than men. Apart from age, smoking, and chronic illness, other factors affecting depression, anxiety, and stress were different in men and women. Investigating the factors associated with depression, anxiety, and stress through a sex-sensitive lens could be helpful for clinicians to diagnose and plan the best therapeutic approach. Mental health policymakers could also better understand the common mental illnesses and take steps to reduce their predictors.
